# The Role of Comorbidities in Predicting 12-Month Survival in Patients with Liver Cirrhosis: A Study from Zahedan, Iran

**DOI:** 10.34172/mejdd.3256

**Published:** 2026-01-31

**Authors:** Hamidreza Zivarifar, Mostafa Ebrahimzadeh, Hamed Taheri, Tahereh Ahrari Roodi

**Affiliations:** ^1^Department of Internal Medicine, and Virology, School of Medicine, Zahedan University of Medical Sciences, Zahedan, Iran; ^2^Clinical Immunology Research Center, Ali-Ebne Abitaleb Hospital, Zahedan University of Medical Sciences, Zahedan, Iran

**Keywords:** Liver cirrhosis, COPD, Blood glucose level, Lipid profile, Survival, Diabetes, COPD, Substance use

## Abstract

**Introduction:**

Liver cirrhosis is a progressive disease characterized by the replacement of healthy liver tissue with scar tissue, leading to impaired liver function. Early diagnosis and management play a crucial role in improving patient outcomes. Comorbidities, including diabetes, hypertension, and dyslipidemia, are known to influence the prognosis of cirrhosis. This study aimed to assess the relationship between comorbid diseases and the 12-month survival of patients with liver cirrhosis in Zahedan, Iran.

**Methods:**

A retrospective cohort of patients with cirrhosis was followed for 12 months. Laboratory indices, including blood glucose, lipid profile, and blood pressure, were monitored. Survival analysis was performed to evaluate the impact of comorbidities on patient survival using statistical methods such as Kaplan–Meier and Cox regression analysis.

**Results:**

Abnormal blood glucose levels, high blood pressure, and dyslipidemia significantly reduced the 12-month survival of patients with cirrhosis. Despite treatment with lipid-lowering medications, lipid disturbances remained a key factor in the survival prognosis. Additionally, factors like body mass index and smoking habits were also identified as influential but often overlooked in clinical practice.

**Conclusion:**

This study underscores the importance of managing comorbidities in patients with cirrhosis to improve survival outcomes. The findings suggest that integrated care addressing both cirrhosis and its associated comorbidities can enhance patient prognosis. Further research is necessary to examine additional factors, such as physical activity, diet, and daily stress levels, which may also affect survival rates.

## Introduction

 Various liver diseases can lead to inflammation and hepatocyte death, a process that ultimately triggers tissue repair and replacement by fibrous (scar) tissue, commonly known as liver cirrhosis.^1^ Histopathological changes in liver cirrhosis involve the substitution of normal liver parenchyma with fibrotic tissue, resulting in irreversible liver damage.^2^ Liver cirrhosis is one of the leading causes of mortality among patients with chronic liver diseases, accounting for 2.9% of all global deaths in 2019 alone.^3^ Major risk factors for liver cirrhosis include hepatitis B infection, hepatitis C infection, alcoholic liver disease, and non-alcoholic fatty liver disease (NAFLD).^4^ However, the influence of comorbid conditions on the clinical course of cirrhosis should not be overlooked. For instance, strong evidence suggests that obesity and diabetes accelerate NAFLD progression, thereby increasing the risk of progression to cirrhosis.^5,6^

 Liver cirrhosis can be classified as compensated or decompensated. Compensated patients are often asymptomatic, with a life expectancy of 9-12 years, though 5-7% develop symptoms annually.^7^ In contrast, decompensated cirrhosis is characterized by life-threatening complications such as portal hypertension, malnutrition, jaundice, hepatic encephalopathy, and even hepatocellular carcinoma, significantly reducing life expectancy. For these patients, liver transplantation remains the only definitive treatment option.^8^ The Child-Turcotte-Pugh (CTP) scoring system is currently the gold standard for assessing cirrhosis severity and predicting patient survival.^9^ This system categorizes patients with cirrhosis into three classes (A, B, and C) based on clinical parameters such as ascites, hepatic encephalopathy, serum albumin and bilirubin levels, and coagulation status (INR). One-year survival rates for these categories range from 45% to 95%.^9,10^

 Recent studies indicate that behavioral factors and comorbid conditions significantly influence the quality of life and survival of patients with cirrhosis.^11^ The presence of multiple comorbidities complicates disease management, disrupts immune and metabolic functions, reduces quality of life, and ultimately increases mortality.^12^ Conditions such as diabetes, hypertension, tuberculosis, and chronic obstructive pulmonary disease (COPD) can adversely affect liver function and patient outcomes through mechanisms such as drug interactions or direct hepatotoxicity, increasing the risk of poor prognosis.^13,14^ Additionally, lifestyle factors such as diet, substance abuse, and alcohol consumption can further exacerbate cirrhosis and shorten survival.^15^ Thus, developing predictive models to assess the impact of comorbidities on disease progression is essential for improving patient management and estimating time to clinical deterioration.^16^ However, current data on the relationship between comorbidities and cirrhosis survival are inconsistent and regionally variable due to differences in environmental factors and healthcare practices.^17^

 In Iran, the prevalence of liver cirrhosis and its complications is on the rise, necessitating a thorough investigation of associated risk factors to inform preventive strategies.^18^ Despite its clinical significance, no comprehensive study has been conducted on this topic. Given the potential benefits of understanding these risk factors for patient management, disease progression analysis, and prognosis, this study aimed to evaluate the impact of comorbidities on 12-month survival in patients with cirrhosis in Zahedan, Iran.

## Materials and Methods

###  Study Design and Sample Size

 This study employed a retrospective cohort survival analysis. Data were extracted from the medical records of patients with liver cirrhosis admitted to Ali ibn Abi Talib Hospital in Zahedan prior to March 21, 2015. From this population, 300 patients were selected and followed for at least one year to ascertain their survival status and related clinical information.

 Given the high prevalence and clinical relevance of COPD, diabetes mellitus, and substance abuse among patients with liver cirrhosis in this region, these three comorbidities were chosen a priori as the focus of analysis. Patients were enrolled into one of three groups: COPD, diabetes mellitus, or substance abuse, with 100 patients assigned to each group. To ensure comparability, sampling was performed using a stratified purposive approach: eligible patients with cirrhosis and one of the three comorbidities were identified from hospital records, and those with complete documentation and at least one year of follow-up were included until each group reached 100 participants. Although this approach limits generalizability to patients with other comorbidities, it allowed for balanced group sizes and focused evaluation of conditions with major implications for cirrhosis prognosis.

 The inclusion criteria were as follows: (1) established diagnosis of liver cirrhosis with at least one of the selected comorbidities (COPD, diabetes mellitus, or substance abuse); (2) fluency in Persian and capacity for verbal communication to enable interviews; (3) provision of informed consent and willingness to participate; and (4) availability of complete clinical examination and follow-up data in hospital records. Patients who did not meet these criteria or declined participation were excluded.

###  Assessment

 For this study, 300 patients with liver cirrhosis were enrolled, consisting of 100 patients in each of the three comorbidity categories: COPD, diabetes mellitus, and substance abuse. Patients were assigned to the group corresponding to their *primary* comorbidity at the time of enrollment. To avoid overlap, each patient was included in only one group, even if multiple comorbidities were present. In such cases, classification was based on the comorbidity judged to have the greatest clinical impact, as determined by the treating hepatologist.

###  Statistical Analysis

 Data were entered into SPSS software version 22 for analysis. The primary endpoint of this study was all-cause mortality within 12 months of enrollment. Survival time was defined as the interval between the date of index hospitalization (i.e., date of cirrhosis diagnosis with one of the selected comorbidities) and either the date of death or the date of censoring. Patients who were alive at 12 months were censored at that time point, and those lost to follow-up before 12 months were censored at the last date on which their vital status was confirmed. Initially, descriptive statistics were reported as medians and interquartile ranges (IQRs) for continuous variables and as frequencies and percentages for categorical variables. The Kaplan–Meier method was used to estimate survival functions, calculate median survival time, and generate survival curves. Group differences in survival were assessed using the log-rank test. To evaluate the effect of demographic, clinical, and potential confounding factors on survival, the Cox proportional hazards regression model was applied to estimate hazard ratios (HRs) and corresponding 95% confidence intervals (CIs).In this study, survival time was defined as the interval between disease diagnosis and the occurrence of complications, and survival times were expressed as median and interquartile range in months. A *P* value of 0.05 was considered statistically significant.

## Results

 In this study, 300 patients with liver cirrhosis who visited Ali ibn Abi Talib Hospital in Zahedan were examined for the presence of three comorbid conditions: COPD, diabetes, and substance use. Among them, 110 patients (36.6%) had COPD, 105 had diabetes, and 120 reported substance use. The demographic characteristics of each group were analyzed separately.

###  Association of COPD with Liver Cirrhosis Survival

 Regarding COPD, 110 patients with cirrhosis (36.6%) were diagnosed with the condition. The demographic distribution of these patients is presented in Table 1. The distribution of laboratory parameters associated with COPD among patients with cirrhosis is summarized in Table 2, revealing significant associations between cholesterol, fasting blood glucose (FBG), two-hour postprandial glucose, systolic blood pressure, body mass index (BMI), HDL, triglycerides, and LDL with the risk of cirrhosis (*P* < 0.05). Specifically, for every 10-unit increase in cholesterol, the risk of cirrhosis increased by 1.005 times (OR = 1.005, 95% CI: 1.002–1.02), while for every 10-unit increase in FBG, the risk increased by 1.01 times (OR = 1.01, 95% CI: 1.01–1.02). Additionally, a 10-unit decrease in HDL was associated with a 1.13-fold increase in the risk of cirrhosis (OR = 1.13, 95% CI: 1.10–1.15).

**Table 1:** Distribution of COPD occurrence in patients with cirrhosis based on demographic variables in Zahedan

**COPD ** **Demographic Variables**		**Yes ** **n (%)**	**No ** **n (%)**
Sex	Female	64 (58.1)	96 (54.0)
	Male	46 (41.9)	94 (49.0)
Occupation	Retired	30 (27.2)	26 (14.0)
	Housewife	36 (32.8)	79 (42.0)
	Self-employed	26 (23.6)	58 (31.0)
	Employee	18 (16.3)	27 (14.0)
Ethnicity	Zaboli	55 (50)	58 (31)
	Baluch	37 (33.6)	82 (43)
	Others	18 (16.3)	50 (26)
Marital status	Divorced/ Single	23 (20.9)	33 (17.0)
	Married	87 (79.1)	157 (83)
Education	Primary	40 (36.3)	56 (29)
	High School	48 (43.6)	58 (31)
	Academic	22 (20.1)	76 (40)
Economic	Low	30 (27.2)	56 (29)
	Medium	58 (52.2)	109 (57)
	High	22 (20.1)	25 (13)
Patient outcome	Death	36 (32.2)	23 (12)
	Under Treatment	74 (67.8)	167 (88)
Age (Mean ± SD)		61.25 ± 46.9	55.42 ± 10.88

**Table 2:** Average laboratory factors examined in patients with liver cirrhosis in Zahedan

**Laboratory Factors**	**COPD**	**Count**	**Mean**	**Standard Deviation**	***P*** ** value**	**OR (%95)**
Cholesterol	Yes	110	157.65	45.67	0.01	1.005 (1.001-1.02)
	No	190	145.36	48.67		
Fasting blood sugar	Yes	110	235.41	59.63	0.01	1.01 (1.01-1.02)
	No	190	156.52	59.63		
Two-hour blood-sugar	Yes	110	304.4	10.5	0.06	1.008 (1.001-1.006)
	No	190	210.53	10.07		
A1c	Yes	110	8.38	2.18	0.02	1.26 (1.15-1.39)
	No	190	7.05	2.42		
Systolic blood pressure	Yes	110	137.14	19.17	0.01	1.052 (1.03-1.06)
	No	190	124.93	12.43		
Body mass index	Yes	110	24.05	2.10	0.02	1.33 (1.22-1.41)
	No	190	21.35	2.05		
Blood lipids	Yes	110	188.61	79.48	0.03	1.005 (1.002-1.008)
	No	190	167.35	57.74		
HDL	Yes	110	34.69	10.17	0.02	1.13 (1.10-1.15)
	No	190	54.05	10.07		
LDL	Yes	110	85.70	32.04	0.01	1.01 (1.005-1.018)
	No	190	74.03	30.98		

 In a multiple logistic regression model examining COPD-related variables in patients with cirrhosis in Zahedan, several factors demonstrated significant associations. Both fasting blood sugar (OR = 1.016, 95% CI: 1.002-1.012, *P* = 0.01) and 2-hour blood sugar (OR = 1.006, 95% CI: 1.008-1.003, *P* = 0.01) were positively associated with COPD. Similarly, age also showed a positive association (OR = 1.06, 95% CI: 1.09-1.036, *P* = 0.03). In contrast, HDL levels were negatively associated with COPD (OR = 0.94, 95% CI: 0.96-0.92, *P* = 0.02). Finally, systolic blood pressure was also positively associated with COPD (OR = 1.03, 95% CI: 1.04-1.01, *P* = 0.01).

 Survival analysis using the Log-Rank test indicated a significant relationship between median survival time (months) in cirrhosis and FBG and 2-hour postprandial glucose levels (*P* < 0.05). Patients with higher fasting and postprandial glucose levels experienced an earlier onset of cirrhosis. In COPD patients with cirrhosis in Zahedan, median survival times varied significantly based on laboratory factors. While cholesterol (*P* = 0.11), A1c (*P* = 0.11), systolic blood pressure (*P* = 0.50), blood lipids (*P* = 0.43), HDL (*P* = 0.50), and LDL (*P* = 0.96) levels showed no significant impact on survival, fasting (*P* = 0.01) and 2-hour blood sugar (*P* = 0.01) were significantly impacted survival, with lower survival times observed when abnormal. The impact of BMI on survival was complex (*P* = 0.72), with no significant difference between the groups. However, there are issues with the confidence interval in the data that need to be resolved before making any conclusions. The cumulative incidence of cirrhosis among patients with COPD was notably higher in those with elevated FBG levels compared with those with normal glucose levels (Figure 1). The final Cox proportional hazards model retained only blood glucose variables as significant predictors. The results of the Cox proportional hazards model indicate that fasting blood sugar is significantly associated with an increased risk of cirrhosis in patients with COPD. The regression coefficient (ß) for fasting blood sugar was 0.01, with a standard error of 0.001, suggesting a precise estimate. The *P* value of 0.01 confirms the statistical significance of this association. The HR for fasting blood sugar was 1.01, indicating that for every unit increase in fasting blood sugar, the risk of cirrhosis increases by 1%. The 95% confidence interval for the HR ranged from 1.01 to 1.08, further supporting the significance of this finding, as the interval does not include the null value of 1. These results suggest that higher fasting blood sugar levels are associated with a slightly elevated risk of cirrhosis in patients with COPD, where each 1-unit increase in FBG corresponded to a 1% increase in the risk of developing cirrhosis.

**Figure 1:**
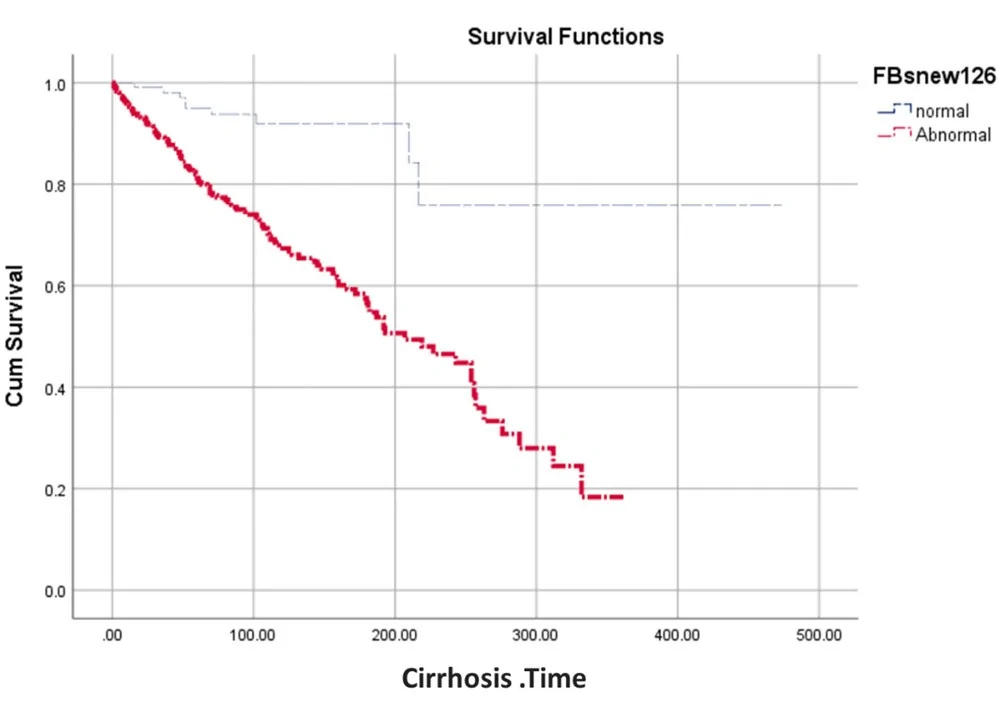
Cumulative incidence of cirrhosis in patients with COPD based on fasting blood glucose

###  Association of Diabetes with Liver Cirrhosis Survival

 With respect to diabetes, 100 patients (33.3%) had both diabetes and cirrhosis, with demographic details provided in Table 3.

**Table 3:** Distribution of concurrent diabetes occurrence based on demographic variables in patients with cirrhosis in Zahedan

**Diabetes ** **Demographic Variables**		**Yes ** **n (%)**	**No ** **n (%)**
Sex	Female	50 (47.4)	102 (52.0)
	Male	55 (52.3)	93 (48.0)
Occupation	Retired	25 (23.8)	52 (27.0)
	Housewife	18 (17.1)	46 (24.0)
	Self-employed	23 (21.9)	63 (32.0)
	Employee	39 (37.1)	34 (17.0)
Ethnicity	Zaboli	30 (28.5)	69 (35)
	Baluch	40 (38)	70 (36)
	Others	35 (33.5)	56 (29)
Marital status	Divorced/ Single	12 (11.5)	15 (8)
	Married	93 (88.5)	180 (92)
Education	Primary	40 (38)	86 (44)
	High School	36 (34.2)	63 (32)
	Academic	29 (27.8)	46 (2418)
Economic	Low	18 (17.1)	56 (29)
	Medium	80 (76.1)	123 (63)
	High	7 (6.68)	16 (8)
Age (Mean ± SD)		58.80 ± 10.2	56.71 ± 11.0

 Laboratory parameters in cirrhotic patients with diabetes are summarized in Table 4, where all variables showed significant associations with cirrhosis (*P* < 0.05). A 10-unit increase in cholesterol increased the risk of cirrhosis by 1.006 times (OR = 1.006, 95% CI: 1.001–1.01), while a 10-unit increase in FBG raised the risk by 1.32 times (OR = 1.32, 95% CI: 1.1–1.71). Additionally, for every 10-unit decrease in HDL, the risk of cirrhosis increased by 1.09 times (OR = 1.09, 95% CI: 1.06–1.11).

**Table 4:** Average laboratory factors examined based on the status of liver cirrhosis occurrence in patients with diabetes in Zahedan

**Laboratory Factors**	**Diabetes**	**Count**	**Mean**	**Standard Deviation**	***P*** ** value**	**OR (%95)**
Cholesterol	Yes	105	167.29	53.43	0.01	1.006 (1.001-1.01)
	No	195	145.82	66.13		
Fasting blood sugar	Yes	105	281.76	71.32	0.02	1.32 (1.002-1.71)
	No	105	179.40	74.89		
Two-hour blood-sugar	Yes	195	270.68	9.5	0.01	1.003 (1.001-1.005)
	No	105	227.68	11.6		
A1c	Yes	195	8.80	1.95	0.01	1.40 (1.25-1.55)
	No	105	6.99	2.40		
Systolic blood pressure	Yes	195	137.49	1.72	0.01	1.04 (1.03-1.06)
	No	105	125.57	1.40		
Body mass index	Yes	195	25.05	3.10	0.02	1.30 (1.21-1.41)
	No	105	22.35	3.05		
Blood lipids	Yes	195	188.41	80.65	0.06	1.005 (1.001-1.008)
	No	105	167.55	58.48		
HDL	Yes	105	42.38	11.10	0.01	1.09 (1.06-1.11)
	No	195	54.11	9.89		
LDL	Yes	105	84.57	35.04	0.09	1.009 (1.002-1.016)
	No	195	75.13	30.21		

 The independent risk factors were further analyzed using multiple logistic regression with the Forward LR method. In a multiple logistic regression model examining diabetes-related variables in patients with cirrhosis in Zahedan, several factors demonstrated significant associations. A1c levels were positively associated with diabetes (OR = 1.21, 95% CI: 1.37-1.08, *P* = 0.01). In contrast, HDL levels were negatively associated with diabetes (OR = 0.93, 95% CI: 0.95-0.91, *P* = 0.01). Finally, systolic blood pressure was also positively associated with diabetes (OR = 1.02, 95% CI: 1.04-1.01, *P* = 0.01).

 In the final Cox proportional hazards model examining diabetes and cirrhosis, A1C, HDL, and systolic blood pressure remained significant (*P* < 0.05). Specifically, a 10-unit increase in A1C was associated with a 1.21-fold increase in cirrhosis risk (OR = 1.21, 95% CI: 1.08–1.37), while a 10-unit increase in HDL corresponded to a 7% decrease in risk (OR = 0.93, 95% CI: 0.91–0.95). Conversely, each 10-unit increase in systolic blood pressure resulted in a 1.02-fold increase in cirrhosis risk (OR = 1.03, 95% CI: 1.01–1.04). The Log-Rank test further demonstrated that median survival time in cirrhosis was significantly influenced by FBG, 2-hour postprandial glucose, A1C, and triglycerides (*P* < 0.05), with earlier cirrhosis onset observed in patients with higher levels of these variables. The analysis of median survival times and Log-Rank test results for diabetes in predicting cirrhosis, stratified by laboratory factors, revealed several key findings. Patients with normal fasting blood sugar levels exhibited a significantly longer median survival time of 296.0 months (95% CI: 263.11–330.83) compared with those with abnormal levels, who had a median survival time of 242.0 months (95% CI: 207.97–277.05; Log-Rank statistic = 1.28, *P* = 0.02). Similarly, patients with normal 2-hour blood sugar levels had a median survival time of 310.92 months (95% CI: 383.38–338.46), which was significantly longer than that of patients with abnormal levels, who had a median survival time of 289.5 months (95% CI: 233.60–297.51; Log-Rank statistic = 15.27, *P* = 0.01). Normal A1c levels were also associated with a significantly longer median survival time of 356.0 months (95% CI: 219.12–292.87) compared with abnormal levels, which had a median survival time of 289.0 months (95% CI: 260.317–45.77; Log-Rank statistic = 2.44, *P* = 0.01). Additionally, patients with normal blood lipid levels had a median survival time of 277.0 months (95% CI: 246.14–308.04), significantly longer than that of patients with abnormal levels, who had a median survival time of 274.44 months (95% CI: 241.84–307.05; Log-Rank statistic = 4.19, *P* = 0.04). Kaplan-Meier survival curves illustrated time-to-cirrhosis progression (Figure 2), showing that patients with higher A1C levels had a greater cumulative incidence of cirrhosis compared with those with normal A1C levels. Similarly, a higher cumulative incidence of cirrhosis was observed in patients with elevated triglyceride levels. The final Cox regression model confirmed FBG, A1C, and 2-hour postprandial glucose as significant predictors of cirrhosis progression. The Cox proportional hazards model analysis identified several independent variables significantly associated with cirrhosis in patients with diabetes. Fasting blood sugar showed a regression coefficient (ß) of 0.004, with a standard error (S.E.) of 0.001 and a *P* value of 0.08. The HR for fasting blood sugar was 1.01 (95% CI: 1.001–1.040), indicating a slight but non-significant increase in the risk of cirrhosis with higher fasting blood sugar levels. In contrast, A1C levels were significantly associated with cirrhosis, with a regression coefficient (ß) of 0.22, a standard error (S.E.) of 0.04, and a *P* value of 0.01. The HR for A1C was 1.25 (95% CI: 1.15–1.36), suggesting a 25% increased risk of cirrhosis for each unit increase in A1C levels. Similarly, 2-hour blood sugar levels were significantly associated with cirrhosis, with a regression coefficient (ß) of 0.003, a standard error (S.E.) of 0.001, and a *P* value of 0.02. The HR for two-hour blood sugar was 1.003 (95% CI: 1.001–1.004), indicating a small but significant increase in the risk of cirrhosis with higher 2-hour blood sugar levels.

**Figure 2:**
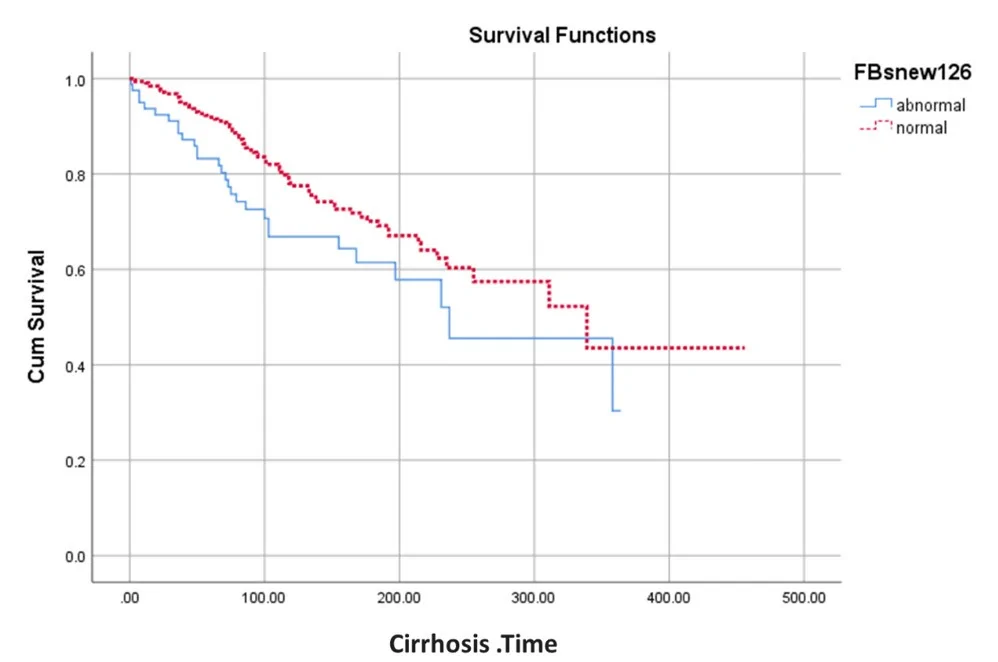
Cumulative incidence of cirrhosis in patients with diabetes based on fasting blood glucose

###  Association of Substance Use with Liver Cirrhosis Survival

 Finally, substance use was reported by 100 patients (33.3%) with cirrhosis. Their demographic characteristics are outlined in Table 5.

**Table 5:** Frequency distribution of drug use based on demographic variables in patients with liver cirrhosis in Zahedan

**Drug Usage ** **Demographic Variables**		**Yes ** **n (%)**	**No ** **n (%)**
Sex	Female	79 (65.8)	87 (48.0)
	Male	41 (34.1)	93 (52.0)
Occupation	Retired	35 (29.1)	51 (28.0)
	Housewife	30 (250)	49 (27.0)
	Self-employed	25 (20.8)	36 (20.0)
	Employee	30 (25)	44 (24.0)
Ethnicity	Zaboli	30 (28.5)	69 (35)
	Baluch	40 (38)	70 (36)
	Others	35 (33.5)	56 (29)
Marital status	Divorced/ Single	116 (96.6)	165 (92)
	Married	4 (3.4)	15 (8)
Education	Primary	46 (38.4)	80 (44)
	High School	49 (40.8)	68 (38)
	Academic	25 (20.8)	32 (18)
Economic	Low	10 (8.2)	39 (22)
	Medium	103 (85.8)	120 (67)
	High	7 (6)	21 (12)
Age (Mean ± SD)		59.26 ± 6.9	56.85 ± 11.0

 Laboratory parameters associated with substance use in patients with cirrhosis revealing significant associations with cholesterol, FBG, and HDL (*P* < 0.05, Table 6). The variables cholesterol, FBG, and HDL demonstrated a statistically significant association with the risk of liver cirrhosis (*P* < 0.05). Specifically, for every 10-unit increase in cholesterol, the odds of developing liver cirrhosis increased by 1.002 times (OR = 1.002, 95% CI: 1.001–1.005). Similarly, a 10-unit increase in FBG was associated with a 1.002-fold increase in the risk of cirrhosis (OR = 1.002, 95% CI: 1.001–1.005). Conversely, a 10-unit decrease in HDL was linked to a 1.13-fold increase in cirrhosis risk (OR = 1.13, 95% CI: 1.10–1.15).

**Table 6:** Mean laboratory parameters in patients with cirrhosis with substance use in Zahedan

**Laboratory Factors**	**Drug Usage**	**Count**	**Mean**	**Standard Deviation**	***P*** ** value**	**OR (%95)**
Cholesterol	Yes	120	256.17	46.40	0.03	1.002 (1.001-1.05)
	No	180	150.28	66.58		
Fasting blood sugar	Yes	120	297.22	77.72	0.05	1.002 (1.001-1.005)
	No	180	236.83	71.14		
Two-hour blood-sugar	Yes	120	304.4	11.42	0.49	1.001 (0.99-1.003)
	No	180	210.53	11.24		
A1c	Yes	120	7.56	2.44	0.68	1.02 (0.91-1.14)
	No	180	7.42	2.32		
Systolic blood pressure	Yes	120	130.37	18.34	0.31	1.00 (0.99-1.02)
	No	180	128.22	15.23		
Body mass index	Yes	120	26.05	2.12	0.32	1.40 (1.21-1.55)
	No	180	21.23	2.04		
Blood lipids	Yes	120	177.68	79.34	0.51	1.001 (0.99 -1.005)
	No	180	171.87	62.39		
HDL	Yes	120	47.64	11.028	0.07	1.13 (0.10-1.15)
	No	180	51.83	12.70		
LDL	Yes	120	79.95	35.33	0.49	1.003 (0.99 -1.01)
	No	180	77.04	31.02		

 To examine independent variables while controlling for potential confounders, multiple logistic regression using the forward likelihood ratio (LR) method was performed. In a multiple logistic regression model examining substance use-related variables in patients with cirrhosis in Zahedan, several factors demonstrated significant associations. Fasting blood sugar was positively associated with substance use (OR = 1.001, 95% CI: 1.004-0.99, *P* = 0.01). Regular follow-up (Yes vs. No) also showed an association with substance use (OR = 1.12, 95% CI: 1.95-0.64, *P* = 0.02). In contrast, HDL levels were negatively associated with substance use (OR = 0.97, 95% CI: 0.99-0.95, *P* = 0.09). Cholesterol was positively associated with substance use (OR = 1.12, 95% CI: 1.95-0.68, *P* = 0.01).

 Regarding the association between drug use and liver cirrhosis, variables such as smoking, FBG, regular medical visits, HDL, and cholesterol remained in the final model (*P* < 0.05). The model indicated that a 10-unit increase in FBG was associated with a 1.001-fold increase in the risk of cirrhosis (OR = 1.001, 95% CI: 0.99–1.004). A 10-unit increase in HDL reduced the risk of cirrhosis by 3% (OR = 0.97, 95% CI: 0.95–0.99). Additionally, individuals with a history of smoking had a 1.12-fold higher risk of cirrhosis compared with non-smokers (OR = 1.12, 95% CI: 0.68–1.95). The analysis of median survival times and Log-Rank test results for patients with liver cirrhosis, stratified by laboratory factors and drug use, revealed several significant findings. Patients with normal fasting blood sugar levels had a significantly longer median survival time of 366.17 months (95% CI: 338.87–393.47) compared with those with abnormal levels, who had a median survival time of 304.14 months (95% CI: 290.83–397.18; Log-Rank statistic = 2.24, *P* = 0.03). Similarly, systolic blood pressure showed a significant association with survival, as patients with normal levels had a median survival time of 371.32 months (95% CI: 345.87–396.77), while those with abnormal levels had a median survival time of 267.0 months (95% CI: 155.69–358.3; Log-Rank statistic = 0.33, *P* = 0.007). Blood lipid levels also demonstrated a significant difference, with normal levels associated with a median survival time of 378.26 months (95% CI: 351.60–404.91) and abnormal levels with 262.70 months (95% CI: 257.23–328.18; Log-Rank statistic = 0.01, *P* = 0.004). In contrast, no significant differences in survival were observed for other laboratory factors. Patients with normal cholesterol levels had a median survival time of 367.27 months (95% CI: 337.57–396.97), while those with abnormal levels had a median survival time of 308.0 months (95% CI: 273.02–344.33; Log-Rank statistic = 0.16, *P* = 0.67). Similarly, two-hour blood sugar levels showed no significant difference, with normal levels associated with a median survival time of 391.55 months (95% CI: 353.48–429.62) and abnormal levels with 351.98 months (95% CI: 322.86–381.10; Log-Rank statistic = 0.28, *P* = 0.59). A1c levels also did not show a significant association, as normal levels were linked to a median survival time of 271.0 months (95% CI: 345.77–396.87) and abnormal levels to 256.0 months (95% CI: 219.12–292.87; Log-Rank statistic = 2.44, *P* = 0.11). Body mass index (BMI) categories similarly showed no significant differences, with thin, normal-weight, and overweight/obese patients having median survival times of 285.0 months (95% CI: 271.08–317.9), 254.0 months (95% CI: 231.08–296.9), and 179.0 months (95% CI: 165.06–267.7), respectively (Log-Rank statistic = 2.34, *P* = 0.82). Additionally, no significant differences were observed for HDL levels, with normal levels associated with a median survival time of 367.69 months (95% CI: 343.53–391.85) and abnormal levels with 278.93 months (95% CI: 206.73–351.12; Log-Rank statistic = 0.59, *P* = 0.44), or for LDL levels, with normal levels associated with a median survival time of 370.0 months (95% CI: 341.38–400.10) and abnormal levels with no reported median survival time (Log-Rank statistic = 0.02, *P* = 0.96. Notably, multicollinearity among independent variables was assessed, and no significant collinearity was detected.

 The Log-Rank test revealed that the median survival time (in months) before cirrhosis onset was significantly associated with regular medical visits (*P* < 0.05), with cirrhosis occurring earlier in individuals who did not adhere to regular medical check-ups. The Cox proportional hazards model analysis identified several independent variables significantly associated with survival outcomes in the study population. Fasting blood sugar showed a regression coefficient (ß) of 0.001, with a standard error (S.E.) of 0.002 and a *P* value of 0.04. The HR for fasting blood sugar was 1.001 (95% CI: 0.996–1.003), indicating a slight but significant increase in risk associated with higher fasting blood sugar levels. Similarly, blood lipids had a regression coefficient (ß) of 0.001, a standard error (S.E.) of 0.002, and a *P* value of 0.04. The HR for blood lipids was 1.001 (95% CI: 0.99–1.002), suggesting a minimal but significant increase in risk with higher blood lipid levels. Regular follow-up was also significantly associated with survival outcomes. Patients who attended regular follow-ups had a regression coefficient (ß) of 0.11, a standard error (S.E.) of 0.25, and a *P* value of 0.02. The HR for regular follow-up was 1.001 (95% CI: 0.99–1.005), indicating a slight but significant reduction in risk for those who maintained regular follow-up compared with those who did not. Furthermore, the Log-Rank test demonstrated significant associations between the median survival time before cirrhosis and FBG, systolic blood pressure, and lipid levels (*P* < 0.05), with earlier cirrhosis onset observed in individuals with elevated levels of these variables.

 Cumulative incidence analysis indicated that among drug users presenting at Ali ibn Abi Talib Hospital in Zahedan, individuals with higher FBG had a greater likelihood of developing cirrhosis compared with those with normal glucose levels (Figure 3). Similarly, those with abnormal systolic blood pressure exhibited a higher cumulative incidence of cirrhosis than individuals with normal systolic blood pressure. Additionally, higher lipid levels were associated with an increased cumulative risk of cirrhosis in drug users compared with those with normal lipid levels.

**Figure 3:**
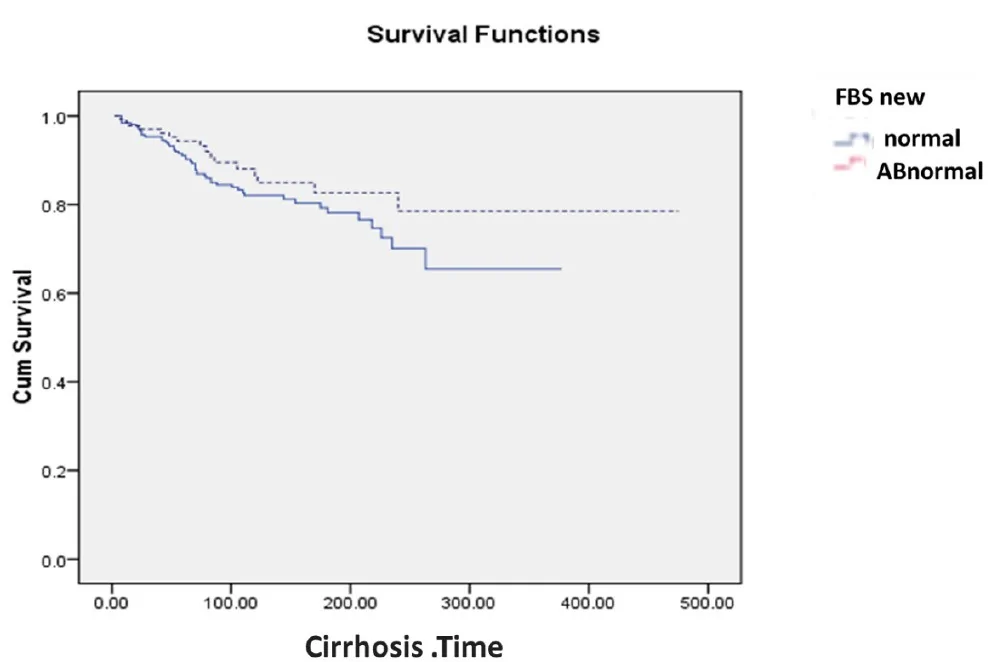
Cumulative incidence of cirrhosis with substance use based on fasting blood glucose

 To identify factors associated with the time to cirrhosis onset while controlling for potential confounders, a multivariate Cox regression model was applied. The final model, selected using the forward LR test. This model incorporates proportionality assumptions and Log-survival plots to analyze the relationship between independent variables and cirrhosis onset. Among patients with cirrhosis presenting to Ali ibn Abi Talib Hospital in Zahedan, only FBG, lipid levels, and regular medical visits remained in the final multivariate Cox regression model. The findings suggest that during the study period, individuals with higher FBG, elevated lipid levels, and irregular medical visits had an increased risk of mortality in cirrhotic patients’ progression.

## Discussion

 Liver cirrhosis is a serious and progressive disease in which healthy liver tissue is gradually replaced by fibrotic tissue, leading to impaired liver function.^19^ Early diagnosis and timely therapeutic interventions play a crucial role in disease management and improving the quality of life in these patients.^20^ Multiple studies have shown that risk factors such as diabetes, heavy substance use, and COPD can significantly impact patient survival.^21-23^ However, no comprehensive study has yet evaluated the influence of underlying conditions on the survival rate of patients with cirrhosis. Therefore, the present study aimed to determine the association between comorbidities and 12-month survival prediction in patients with cirrhosis in Zahedan.

 According to this study’s findings, most laboratory indicators, including blood glucose, lipid profile, and blood pressure, were outside the normal range among the studied patients. Survival analysis demonstrated that abnormal blood glucose levels significantly reduced survival across all groups. Consistent with our findings, Garcia et al. (2010) reported that abnormal blood glucose levels exacerbate the severity and complications of cirrhosis, leading to increased mortality in affected patients.^22^

 We observed that, despite recognizing diabetes and COPD as major risk factors, other contributing factors, such as hypertension, BMI, and smoking, have been largely overlooked in the management of patients with cirrhosis. Moreover, despite the use of lipid-lowering medications, dyslipidemia remained prevalent among patients with cirrhosis, adversely affecting survival indices. Tripodi and other found that patients with cirrhosis who regularly attended medical check-ups had better laboratory profiles and, consequently, improved survival rates.^23^

 Hemoglobin A1C was identified as another key factor influencing the quality of life and survival of patients with cirrhosis. Our results demonstrated that elevated A1C levels were significantly associated with reduced survival in patients with cirrhosis. In this regard, Wiegand’s study showed that even a 1% reduction in A1C could lead to a 21% decrease in cirrhosis-related complications, disabilities, and mortality.^24^ Similarly, findings by Wanless and colleagues confirmed a direct relationship between A1C levels and the survival duration of patients with cirrhosis.^25^

 Our study demonstrated that several comorbidities, particularly diabetes mellitus and COPD, were strongly associated with reduced 12-month survival in patients with cirrhosis. Abnormal blood glucose levels and elevated hemoglobin A1C emerged as key predictors of poor prognosis, consistent with previous reports showing that hyperglycemia worsens cirrhosis complications and increases mortality.^22-25^ Similarly, COPD contributed to decreased survival, highlighting the importance of cardiopulmonary health in patients with cirrhosis.

 Beyond these major comorbidities, additional risk factors such as hypertension, dyslipidemia, increased BMI, and smoking also contributed to unfavorable survival outcomes. Despite the use of lipid-lowering therapies, dyslipidemia remained common. It adversely affected survival indices, supporting Tripodi et al.’s observation that patients under regular medical supervision tend to achieve better laboratory control and survival.^23^ These findings suggest that comprehensive management of metabolic and cardiovascular risk factors should be prioritized in patients with cirrhosis.

 In this study, no significant association was found between substance use and survival in patients with cirrhosis. This result contrasts with several previous studies that reported substance use, particularly alcohol and opioids, as major contributors to cirrhosis progression and mortality. ^26,28,29^ A possible explanation for the lack of statistical significance in our cohort may relate not only to the relatively limited sample size, but also to underreporting of substance use due to social stigma, which is especially relevant in our cultural context. Additionally, many patients in our study were already in advanced stages of cirrhosis, where other comorbidities such as diabetes and COPD may have overshadowed the impact of substance use on short-term (12-month) survival outcomes. Another explanation could be that some patients were already receiving medical care and abstaining from active substance use at the time of enrollment, thereby reducing its direct effect on survival. Nevertheless, our findings should not be interpreted as diminishing the importance of substance use in cirrhosis prognosis. Rather, they underscore the complexity of interactions between substance use and other comorbid conditions. Further longitudinal studies with larger sample sizes, more detailed data on the type, duration, and intensity of substance use, and longer follow-up periods are needed to better elucidate its role in survival outcomes.

 Given the high prevalence of cirrhosis, identifying key factors influencing disease prognosis is essential, particularly in developing countries like Iran, where such aspects have received less attention.^27^ This study highlighted the significant role of comorbid conditions in determining survival rates in patients with cirrhosis. Effective prevention and management of these underlying conditions can greatly improve both quality of life and survival outcomes in patients with cirrhosis. Our findings underscore the need for increased efforts and strategic planning to enhance healthcare services, including education and preventive interventions, to reduce cirrhosis-related complications.

 This study provides valuable insights into the role of comorbidities in predicting the 12-month survival of patients with liver cirrhosis. One of its key strengths is the comprehensive analysis of multiple metabolic and clinical factors, including diabetes, hypertension, dyslipidemia, and COPD, which have often been overlooked in cirrhosis management. Additionally, the use of survival analysis enhances the reliability of our findings in assessing the long-term impact of these conditions on patient outcomes. Furthermore, by focusing on a population in Zahedan, this study contributes to the limited data available on patients with cirrhosis in developing countries, thereby filling an important gap in regional epidemiological research. Despite these strengths, the study has several limitations. First, it relied on patients’ most recent laboratory test results, without accounting for dynamic changes in biochemical markers over time, which may have provided a more accurate assessment of disease progression. Second, although key comorbidities were analyzed, other potentially influential factors, such as physical activity, dietary patterns, quality of life, and psychological stress, were not included. Third, the sample size was relatively small, which may have limited the statistical power to detect associations with certain variables, such as substance use. Lastly, given the study’s observational design, causality cannot be established, and further longitudinal studies are needed to validate these findings.

## Conclusion

 In conclusion, our study highlights the significant role of comorbidities in predicting the 12-month survival of patients with liver cirrhosis in Zahedan. Factors such as abnormal blood glucose levels, hypertension, dyslipidemia, and diabetes were found to be crucial predictors of survival. These findings emphasize the importance of early diagnosis and comprehensive management of comorbid conditions to improve the prognosis of patients with cirrhosis. Despite some limitations, such as the exclusion of certain influencing factors, such as physical activity and dietary habits, this study underscores the need for more targeted interventions and better clinical management strategies to enhance the quality of life and survival rates in patients with cirrhosis. Further research with larger sample sizes and a broader range of variables is required to confirm these results and guide evidence-based practices in cirrhosis care.
